# Multidrug-Resistant Tuberculosis in Central and Eastern Europe: Implementation and Maturity of Whole-Genome Sequencing for Surveillance

**DOI:** 10.3390/diseases14050172

**Published:** 2026-05-14

**Authors:** Dragos Baiceanu, Laura Ioana Chivu, Roxana-Mihaela Coriu, Alexandru Stoichita, Traian-Constantin Panciu, Dragos-Cosmin Zaharia, Beatrice Mahler, Anca Matei, Elmira Ibraim, Loredana Sabina Cornelia Manolescu

**Affiliations:** 1 Doctoral School, Faculty of Medicine, Carol Davila University of Medicine and Pharmacy, 020021 Bucharest, Romania; dragos.baiceanu@drd.umfcd.ro (D.B.); alexandru.stoichita@drd.umfcd.ro (A.S.); traian-constantin.panciu@drd.umfcd.ro (T.-C.P.); 2 Department of Research, Marius Nasta Institute of Pneumology, 050159 Bucharest, Romania; ielmira2000@yahoo.com; 3 Department of Pathophysiology, Faculty of Midwifery and Nursing, Carol Davila University of Medicine and Pharmacy, 020021 Bucharest, Romania; 4 Clinical Laboratory of Medical Microbiology, Marius Nasta Institute of Pneumology, 050159 Bucharest, Romania; loredana.manolescu@umfcd.ro; 5 Department of Cardio-Thoracic Pathology, Faculty of Medicine, Carol Davila University of Medicine and Pharmacy, 020021 Bucharest, Romania; dragos.zaharia@umfcd.ro (D.-C.Z.); beatrice.mahler@umfcd.ro (B.M.); 6 Department of Pulmonology, Marius Nasta Institute of Pneumology, 050159 Bucharest, Romania; 7 Department of Endocrinology, Faculty of Medicine, Grigore T. Popa University of Medicine and Pharmacy, 700115 Iasi, Romania; anca.matei@umfiasi.ro; 8 Department of Fundamental Sciences, Faculty of Midwifery and Nursing, Carol Davila University of Medicine and Pharmacy, 020021 Bucharest, Romania

**Keywords:** multidrug-resistant tuberculosis, infectious diseases, genomic surveillance, public health surveillance, molecular epidemiology, drug resistance, transmission, Central and Eastern Europe

## Abstract

Background/Objectives: Multidrug-resistant tuberculosis (MDR-TB) remains a major public health challenge in the WHO European Region, which reports the highest global proportion of rifampicin-resistant and MDR-TB cases. Whole-genome sequencing (WGS) has emerged as a key tool for improving drug-resistance detection and supporting molecular surveillance. However, the level of genomic implementation across Central and Eastern Europe (CEE) remains insufficiently characterized. This scoping review aimed to evaluate the use of WGS for MDR-TB in CEE countries and to classify implementation maturity using a predefined framework (L0–L4). Methods: A structured search of PubMed/MEDLINE and Web of Science identified original studies published in English between 2015 and 2026 reporting genomic applications in MDR-TB across 13 predefined CEE countries. Data were extracted on sequencing approaches, resistance prediction, transmission analysis, monitoring of new or repurposed drugs, bioinformatic pipelines, and programmatic integration. Countries were categorized according to a five-level maturity model based on documented capacity, scope of application, and integration into national tuberculosis programs (NTPs). Results: Twenty-eight studies were included. WGS was used in 23/28 studies (82.1%), predominantly for genomic resistance prediction (25/28). Transmission analysis was reported in 19/28 studies, with heterogeneous single nucleotide polymorphism (SNP) thresholds and clustering methodologies. Monitoring of resistance to new or repurposed drugs was described in 8/28 studies. No country achieved Level L4 (formally integrated genomic surveillance). Four countries were classified as L3 and nine as L2, while no L0 or L1 settings were identified. Conclusions: Countries in Central and Eastern Europe demonstrate increasing operational use of WGS for MDR-TB, primarily driven by clinical resistance prediction. However, the lack of formal integration into national surveillance systems highlights a persistent gap between technological adoption and structured public health implementation. Strengthening programmatic integration and methodological standardization is essential for advancing genomic surveillance of MDR-TB in the region.

## 1. Introduction

Tuberculosis (TB) continues to be a major global public health problem, and drug-resistant forms, particularly multidrug-resistant tuberculosis (MDR-TB), pose one of the most serious threats to disease control. According to the latest estimates from the World Health Organization (WHO), the European Region has the highest global proportion of rifampicin-resistant tuberculosis (RR-TB) or MDR-TB cases. In some Eastern European countries, more than 20% of new cases and more than 50% of previously treated cases are RR-TB or MDR-TB [1]. At the same time, treatment success rates for MDR-TB and extensively drug-resistant (XDR-TB) forms remain significantly lower than for drug-sensitive TB [1]. These epidemiological patterns highlight the need for strengthened disease control strategies, particularly in early diagnosis, resistance detection, and surveillance systems.

Traditionally, drug resistance has been attributed predominantly to selection under inadequate or incomplete treatment. However, molecular and phylogenetic evidence accumulated over the last decade indicates that primary transmission of already resistant strains contributes substantially to the burden of MDR-TB in certain regions. Large-scale genomic studies have demonstrated the existence of MDR clades with increased epidemic success, capable of transnational spread and progressive accumulation of additional resistance [2]. A relevant example is the W148 (European/Russian) clade, associated with widespread epidemics in Eastern Europe and Central Asia and with documented evolution towards pre-XDR and XDR forms [2]. These findings emphasize that MDR-TB represents not only a therapeutic challenge, but also a major issue for transmission dynamics and public health control at the population level.

The implementation of whole-genome sequencing (WGS) has fundamentally changed the approach to tuberculosis diagnosis and surveillance. WGS allows for the rapid and comprehensive identification of drug resistance mutations, overcoming the limitations of conventional phenotypic tests, which are slow and, for some second-line drugs, insufficiently standardized [3]. Correlating mutations detected by WGS with minimum inhibitory concentration (MIC) values has shown that different genetic variants can confer distinct levels of resistance, with potential impact on therapeutic decisions and treatment regimen optimization [3]. In this context, WGS has become increasingly relevant as a tool supporting both clinical management and public health surveillance of MDR-TB.

Beyond resistance prediction, WGS offers superior resolution for transmission analysis and genomic cluster identification. Recent European initiatives have demonstrated the feasibility of using genomic data in cross-border surveillance of MDR-TB. The EuSeqMyTB pilot project, coordinated by the European Centre for Disease Prevention and Control (ECDC), analyzed over 2000 RR/MDR-TB isolates from 25 European Union/European Economic Area (EU/EEA) countries, identifying international clusters and highlighting the need for methodological harmonization and interoperability between laboratories [4]. Subsequently, defining SNP panels for major MDR clades were developed to facilitate the integration of genomic screening into routine workflows [5]. However, these initiatives highlighted significant heterogeneity in sequencing capacity, bioinformatics infrastructure, and the level of institutional integration between European countries.

In parallel, phylodynamic approaches have allowed for exploration of the relationship between resistance mutations and the transmissibility of MDR strains. Models estimating the fitness costs associated with resistance suggest that certain compensatory mutations may reduce the biological impact of resistance and facilitate sustained transmission [6]. These results underscore the fact that genomic surveillance must go beyond the simple identification of mutations and include their interpretation in the context of population dynamics.

The Central and Eastern European (CEE) region occupies a strategic position in this epidemiological landscape. Several countries in the region contribute significantly to the regional burden of MDR-TB, and the transnational circulation of resistant clades has been repeatedly documented [2]. At the same time, the implementation of WGS in CEE is heterogeneous, ranging from limited use in research projects to repeated application for resistance prediction and transmission analysis. Although there are national studies and regional initiatives, there is a lack of systematic and comparative assessment of the maturity level of genomic implementation in this region. This gap limits the understanding of how genomic approaches contribute to disease control and surveillance capacity across countries.

Therefore, this scoping review aims to assess the use of WGS for MDR-TB in Central and Eastern European countries and to classify the level of implementation using a predefined maturity framework (L0–L4). The objective was to characterize its use for resistance prediction, transmission analysis, and programmatic integration, as well as the existence of a sustained internal sequencing capacity. By addressing these aspects, the study aims to provide a disease-oriented and public health-relevant perspective on the current state of genomic surveillance for MDR-TB in the region. Through this approach, the study provides a comparative perspective on the current state of genomic implementation in the region and helps clarify its position in relation to the formal integration of genomic surveillance into national tuberculosis control programs.

## 2. Material and Methods

### 2.1. Study Design

**Figure 1:**
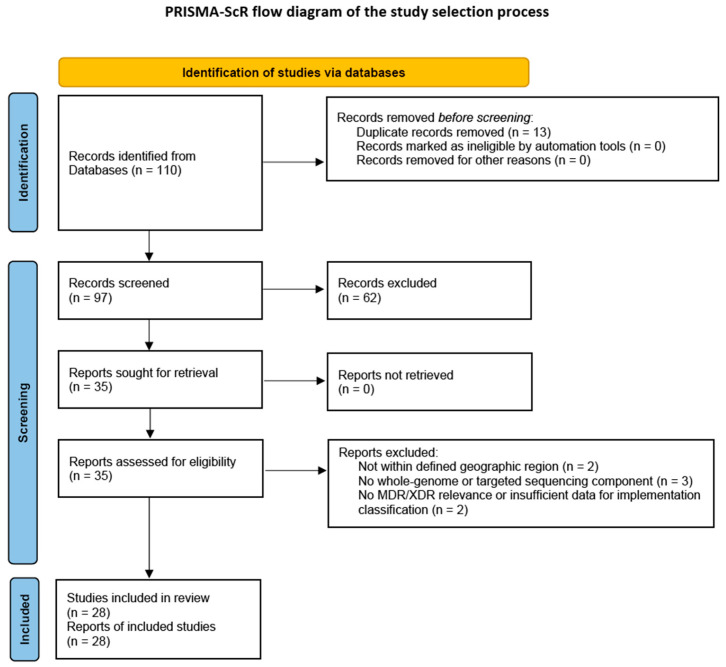
PRISMA-ScR flow diagram of the study selection process. Records were excluded during title and abstract screening (*n* = 62) based on predefined eligibility criteria, including studies conducted outside the defined CEE region, absence of a genomic sequencing component, case reports without comparative genomic analysis, purely methodological studies without epidemiological applicability, and studies not focused on MDR/XDR-TB or genomic surveillance. Adapted from Page et al. [8].

This study was conducted as a scoping review, developed in accordance with the PRISMA-ScR guideline [7] (Preferred Reporting Items for Systematic Reviews and Meta-Analyses—extension for Scoping Reviews). A completed PRISMA-ScR checklist is provided in the Supplementary Materials, and the study selection process is illustrated in Figure 1.

The choice of this methodological design was determined by the substantial heterogeneity of the literature on the implementation of WGS and related genomic approaches for MDR-TB surveillance in Central and Eastern Europe. The studies identified differ in terms of the technologies used (WGS, targeted sequencing, different genetic panels), the objectives analyzed (prediction of drug resistance, transmission analysis, investigation of outbreaks), selective sampling versus national surveillance approaches, and the level of clinical and programmatic integration.

Given this methodological variability and the lack of standardized coverage and integration indicators, conducting a quantitative meta-analysis would not have been appropriate. Instead, the scoping review approach allowed for:

• Systematic mapping of the relevant literature;
• Comparison of implementation models across countries;
• Identification of regional gaps;
• Development of a conceptual framework for assessing implementation maturity.

The study protocol was not pre-registered. Although the protocol was not prospectively registered, the review followed predefined eligibility criteria, search strategies, and classification rules established prior to data extraction.

### 2.2. Region Definition

The analysis was limited to a predefined group of countries in Central and Eastern Europe, selected on the basis of geographical proximity, epidemiological continuity, and relevance to the context of tuberculosis surveillance in Romania.

The following countries were included: Poland, the Czech Republic, Slovakia, Hungary, Romania, Bulgaria, Croatia, Slovenia, Estonia, Latvia, Lithuania, the Republic of Moldova, and Ukraine.

European Union member states were included based on their membership in the Central and Eastern European region according to geographical classifications used in international literature. The Republic of Moldova and Ukraine were included due to regional epidemiological continuity, the high prevalence of MDR-TB, and the existence of documented cross-border transmission, which is directly relevant to Romania’s health security.

The Russian Federation and the Central Asian states were not included in order to maintain a coherent and comparable regional delimitation in terms of infrastructure and programming.

### 2.3. Search Strategy and Data Sources

The literature search was conducted in two bibliographic databases: PubMed/MEDLINE and Web of Science Core Collection, in accordance with PRISMA-ScR recommendations for scoping reviews.

The PubMed search was performed on 10 February 2026, and covered the period 1 January 2015 to 10 February 2026.

The search in the Web of Science Core Collection was performed on 11 February 2026 and covered the period 1 January 2015 to 31 December 2025, according to the filtering options available on the platform.

#### 2.3.1. Strategy Used in PubMed

The search was performed using the following string of terms combined by Boolean operators: (“whole genome sequencing” OR WGS OR “next generation sequencing” OR “targeted sequencing” OR “amplicon sequencing” OR “genomic surveillance” OR “molecular epidemiology”) AND (“*Mycobacterium tuberculosis*” OR tuberculosis OR TB) AND (“multidrug resistant” OR MDR OR XDR OR “drug resistant”) AND (“Central Europe” OR “Eastern Europe” OR Poland OR Romania OR Bulgaria OR Slovakia OR Czech OR Hungary OR Estonia OR Latvia OR Lithuania OR Moldova OR Ukraine).

#### 2.3.2. Strategy Used in Web of Science Core Collection

The search was performed in the “Topic” field (title, abstract, keywords), using the following expression: (“whole genome sequencing” OR WGS OR “next generation sequencing” OR “targeted sequencing” OR “amplicon sequencing” OR “genomic surveillance” OR “molecular epidemiology”) AND (“*Mycobacterium tuberculosis*” OR tuberculosis) AND (“multidrug resistant” OR MDR OR XDR OR “drug resistant”) AND (Poland OR Romania OR Bulgaria OR Slovakia OR Czech OR Hungary OR Estonia OR Latvia OR Lithuania OR Moldova OR Ukraine).

Variations in terminology (e.g., “multidrug-resistant”, “multi-drug resistant”) were considered to be captured through the use of broader terms such as “MDR” and “drug resistant”.

The following filters were applied: document type: Article; language: English; time range: 1 January 2015–31 December 2025 for Web of Science Core Collection, and 1 January 2015–10 February 2026 for PubMed. The results were exported in tabular format and subsequently analyzed in Microsoft Excel for the selection process. Grey literature sources and conference abstracts were not included.

### 2.4. Study Selection

The selection of studies was carried out in several consecutive stages. All records identified through database searches were exported and centralized in a Microsoft Excel © file.

In the first stage, the titles and abstracts were screened to exclude obviously irrelevant studies, including articles from outside the defined region, studies without a genomic sequencing component, isolated case reports, or methodological studies without epidemiological applicability. Subsequently, duplicates between databases were identified and eliminated by comparing titles, authors, and year of publication.

The remaining articles were evaluated by analyzing the full text to confirm eligibility according to predefined criteria. At this stage, seven articles were excluded for methodological or regional relevance reasons, resulting in a final total of 28 studies included in the scoping review analysis.

The selection process is presented in the PRISMA-ScR flow diagram (Figure 1).

### 2.5. Eligibility Criteria

Original studies that cumulatively met the following criteria were included in the analysis:

• Studies published between 2015 and 2026;
• Studies written in English;
• Studies that reported the use of genomic sequencing (whole genome sequencing or targeted sequencing) for *Mycobacterium tuberculosis*;
• Studies that included data from one or more of the countries defined in the regional scope of the study;
• Studies that were relevant to multidrug-resistant tuberculosis (MDR-TB), pre-extensively drug-resistant tuberculosis (pre-XDR-TB), or extensively drug-resistant tuberculosis (XDR-TB), including genomic surveillance, transmission, or resistance prediction analyses.

The following were excluded:

• Studies conducted outside the defined region;
• Articles without a genomic sequencing component;
• Purely methodological studies without epidemiological applicability;
• Isolated case reports without comparative genomic analysis;
• Clinical or therapeutic studies without associated genomic analysis;
• Review, editorial, or commentary articles.

No additional restrictions were applied to the study design (retrospective, prospective, outbreak analysis, national surveillance), as the purpose of the scoping review was to map the use and level of implementation, not to evaluate the effectiveness of interventions.

### 2.6. Data Extraction

The data from the included studies were manually extracted and centralized in a standardized table in Microsoft Excel, developed prior to the analysis process.

The following information was collected for each study:

• Lead author and year of publication;
• Country or countries from which the analyzed data originated;
• Type of study (outbreak investigation, genomic characterization of isolates, pilot implementation, national surveillance, methodological analysis);
• Number of isolates analyzed and collection period;
• Type of cases included (MDR, pre-XDR, XDR, or all forms of TB);
• Type of sequencing used (whole genome sequencing from culture, direct sequencing from sputum, targeted sequencing);
• Sequencing platform;
• Bioinformatics tools or pipeline used for data analysis;
• SNP threshold used to define transmission clusters, where specified;
• Use of WGS-based analysis to investigate transmission;
• Use of WGS-based analysis to predict drug resistance;
• Assessment of resistance to new drugs (bedaquiline, linezolid, delamanid, pretomanid);
• Level of implementation of genomic sequencing in the national context (according to the L0–L4 framework).

Data were extracted consistently for all included studies, and any missing information was explicitly marked. Data extraction was conducted by one reviewer using a predefined standardized form developed prior to analysis. No duplicate independent charting was performed and authors of the original studies were not contacted for missing information.

### 2.7. Framework for Assessing the Maturity of WGS Implementation (L0–L4)

To enable a comparative analysis between the countries included, a conceptual framework for assessing the maturity of WGS implementation for MDR-TB was developed, structured on five progressive levels (L0–L4), where “L” indicates the level of implementation.

The model was developed inductively based on the literature reviewed and reflects patterns of WGS implementation reported in the region. While conceptually informed by previous European work on genomic surveillance, the framework represents an original analytical model adapted to the regional context of MDR-TB genomic capacity in Central and Eastern Europe.

The assessment was based on four operational areas:

• Existence of local sequencing capacity;
• Punctual or repetitive nature of use;
• Purpose of application (resistance prediction and/or transmission analysis);
• Degree of clinical and programmatic integration.

The classification was based exclusively on information explicitly reported in the included studies. In the absence of clear evidence of clinical or programmatic integration, no assumptions were made, and a conservative approach was applied.

• L0—Absence of local capacity;

No operational local capacity for WGS or targeted sequencing is described. Sequencing is performed exclusively through external collaborations or on an ad hoc basis, with no reported local infrastructure or bioinformatics pipeline.

• L1—Activity limited to research;

Sequencing is performed locally or in collaboration, but exclusively within specific studies. There is no repetitive use or clinical/programmatic integration. Activity is limited to small batches and no operational continuity is described.

• L2—Clinical implementation for resistance prediction;

Sequencing is used repeatedly to identify mutations associated with drug resistance, with reported clinical impact.

Minimum criteria: Recurrent use (not just a single study), described genetic panel or bioinformatics pipeline, use to support therapeutic decisions or confirm drug susceptibility testing (DST), partial integration into the clinical workflow.

Transmission analyses may be reported on an ad hoc basis or performed through external collaborations; however, there is no evidence of repetitive use for transmission surveillance, sustained internal capacity, or systematic implementation at the national level.

• L3—WGS used for selective surveillance and transmission analysis;

WGS is used repeatedly to investigate transmission and identify clusters, supported by operational national capacity or systematic coordination, at least for MDR/XDR cases or in the context of outbreaks.

Minimum criteria: Explicit use of SNP thresholds or phylogenetic analyses, correlation with epidemiological data, described bioinformatic pipeline, documented use in more than one study or distinct period, evidence of internal capacity or coordinated mechanism for transmission analysis, integration into the national program may be partial.

• L4—Nationally integrated genomic surveillance;

Genomic sequencing is formally integrated into the national surveillance system for MDR-TB.

Minimum criteria: National criteria defined for sequencing, systematic use for MDR/XDR cases, national reporting mechanism or genomic database, explicit integration into the NTP.

Application and aggregation rules: Classification was initially performed at the individual study level. Subsequently, the final level for each country was determined based on the highest level for which there was consistent evidence of repetitive implementation and sustained capacity. In the event of discrepancies between studies, a conservative classification approach was applied, meaning that the level of implementation was assigned only when supported by consistent and explicit evidence, avoiding overestimation of national capacity. The proposed model represents an analytical framework derived from the available literature and not an officially validated standard.

### 2.8. Data Analysis and Synthesis

Data analysis was descriptive and comparative. The unit of analysis was the individual study. No meta-analysis was performed, given the methodological heterogeneity of the included studies. No formal assessment of bias risk was performed, as this type of analysis is not mandatory in a scoping review. When evidence was limited to a single study, classification was performed conservatively and should be interpreted as indicative rather than definitive. A small number of studies using classical molecular typing methods were retained to provide contextual epidemiological information and to reflect the transition toward genomic approaches in the region; however, these studies were not considered as primary evidence for WGS-based implementation levels.

For each study, the following information was synthesized:

- The type of sequencing used;
- The purpose of application (resistance prediction, transmission, surveillance);
- The SNP thresholds used for clustering (where applicable);
- The assessment of resistance to new and repurposed drugs;
- The preliminary level of implementation (L0–L4).

1. Country-level aggregation: after individual classification of the studies, the final maturity level for each country was determined by aggregation based on the 28 studies included in the final analysis.

- If there was consistent evidence of repetitive implementation and integration, the country was classified at the level corresponding to the highest level supported by the data.
- Isolated or pilot studies were not considered sufficient for reclassification to a higher level.
- In the event of discrepancies between studies, a conservative approach was applied.

2. Primary outcomes: two main outcomes of interest were defined:

- Level of maturity of genomic sequencing implementation (L0–L4) per country.
- Predominant type of sequencing use (research, resistance prediction, transmission investigation, surveillance).

3. Secondary outcomes: the following were additionally analyzed:

- Genomic monitoring of resistance to new drugs (bedaquiline, linezolid, clofazimine, delamanid, pretomanid);
- SNP thresholds used to define clusters;
- Dominant lineages and epidemic clades described.

4. Data presentation: results are presented:

- In tabular form (study characteristics and classification by country);
- Narratively, through comparative synthesis between countries;
- Graphically (PRISMA-ScR diagram and, optionally, schematic representation of L0–L4 levels).

No inferential statistical analyses were performed.

## 3. Results

### 3.1. Study Selection

The study selection process is summarized in Figure 1.

The database search identified a total of 110 records (75 from PubMed/MEDLINE and 35 from Web of Science Core Collection). Prior to screening, 13 duplicates were removed, leaving 97 records for title and abstract assessment.

Following screening, 62 records were excluded based on predefined eligibility criteria (see Figure 1). Thus, 35 articles were considered eligible for full assessment.

At this stage, 7 articles were excluded for the following reasons: studies conducted outside the defined region (*n* = 2), absence of a genomic sequencing component (WGS or targeted sequencing) (*n* = 3), and lack of relevance to MDR/XDR-TB or insufficient data for classification of the level of implementation (*n* = 2). Finally, 28 studies met all inclusion criteria and were included in the descriptive analysis of this scoping review.

### 3.2. Characteristics of the Included Studies

The 28 included studies were published between 2015 and 2026 (median: 2021), with a noticeable increase in the number of publications after 2018 and a relatively constant rate between 2020 and 2023 (four studies per year in 2020, 2021, and 2022). Twenty-four studies were identified through PubMed/MEDLINE and four through Web of Science. A detailed presentation of the characteristics of the included studies is available in Table 1 [3,9,10,11,12,13,14,15,16,17,18,19,20,21,22,23,24,25,26,27,28,29,30,31,32,33,34,35].

The studies covered all 13 countries included in the region, including both national analyses and multicenter or cross-border investigations. Because some papers analyzed cohorts from multiple jurisdictions, a single study could contribute data for multiple countries; therefore, the number of national contributions may exceed the total of 28 studies.

WGS was used in 23/28 studies (82.1%), predominantly on isolates obtained from culture. One study applied targeted NGS sequencing exclusively, and four used classical molecular methods (MIRU-VNTR, spoligotyping, or other PCR-based techniques) without whole genome analysis. Illumina platforms were dominant; MiSeq was reported in 12 studies, NextSeq in 3, NovaSeq in 1, and in seven studies the exact platform model was not specified.

A bioinformatic pipeline was mentioned in 25/28 studies (described in full in 23 and in part in 2), while three studies did not report details on bioinformatic analysis. Of the 19 studies that evaluated genomic transmission or clustering, SNP thresholds were heterogeneous; in 11 cases, no clear threshold was specified, and in the rest, thresholds ≤5 SNP (*n* = 6) or 12 SNP (*n* = 3) were most commonly used, with alternative values reported occasionally.

Transmission analysis was reported in 19/28 studies, genomic prediction of drug resistance in 25/28, and assessment of resistance to new or repurposed drugs (e.g., bedaquiline, linezolid, clofazimine, delamanid) in 8/28 studies.

Regarding integration into national tuberculosis control programs, integration was reported as present in 6/28 studies, partial in 3/28, absent in 11/28, and unspecified in 8/28, suggesting a variable degree of formalization of WGS use in routine surveillance.

### 3.3. Genomic Implementation Maturity (L0–L4)

Applying the L0–L4 maturity model defined in the Section 2 (Material and Methods), the 13 countries included showed varying levels of implementation of genomic sequencing for MDR-TB. The classification was performed at the country level based on documented use of WGS for resistance prediction, transmission analysis, and degree of programmatic integration, including the existence of sustained in-house capacity. Country-level maturity classification is summarized in Table 2.

#### Distribution of Maturity Levels

The classification was based solely on information explicitly reported in the included studies. In the absence of clear evidence of clinical or programmatic integration, no assumptions were made, and a conservative approach was taken.

- L0—Absence of documented capacity (*n* = 0)

No country was identified without documented capacity to use genomic sequencing during the period analyzed.

- L1—Research-only implementation (*n* = 0)

No countries were identified where the use of WGS was strictly limited to isolated academic projects, without applications in resistance prediction or epidemiological analysis.

- L2—Limited clinical integration (*n* = 9 countries)

Nine countries were classified at level L2: Slovakia, Hungary, Romania, Bulgaria, Croatia, Slovenia, Lithuania, Ukraine, and Estonia. In these contexts, WGS was mainly used for drug resistance prediction, and transmission analysis was absent, limited, or performed on an ad hoc basis, often through external collaborations. Internal sequencing capacity and formal integration into NTP were not documented.

In some countries (e.g., Ukraine and Lithuania), the use of WGS for transmission was reported on an ad hoc basis, with no evidence of repeated application in surveillance, which is why they were conservatively classified at level L2. In the case of Romania, WGS was predominantly used through outsourcing to international centers, with no evidence of an operational national infrastructure or systemic integration into routine surveillance, which is why it was classified at level L2.

- L3—Use for transmission and selective surveillance (*n* = 4 countries)

Four countries have reached level L3: Poland, the Czech Republic, Latvia, and the Republic of Moldova. In these cases, WGS has been used repeatedly for transmission analysis and identification of genomic clusters, indicating an advanced level of epidemiological applicability. Some countries demonstrated strengthened internal capacity and the extension of analyses to new or repurposed drugs.

The distinction between the Republic of Moldova and Romania was based on the repeated and coordinated use of WGS for transmission and clustering analyses in Moldova, whereas in Romania the available evidence mainly reflected externally supported pilot or outbreak investigations without documented recurrent national use for transmission surveillance.

Although the Republic of Moldova reported systematic use of WGS at the national level during the periods analysed, in the absence of formal documentation of an integrated genomic surveillance policy within the NTP, it was not classified at level L4 according to the strict criteria defined above.

- L4—Formally integrated genomic surveillance (*n* = 0)

No country in the region met the criteria for level L4, which requires the formal and institutionalized integration of genomic surveillance into the NTP.

Overall, all 13 countries analyzed are at intermediate levels (L2–L3), reflecting a gradual transition toward the use of genomics in MDR-TB surveillance, but without systematic and formalized implementation at the national level.

**Figure 2:**
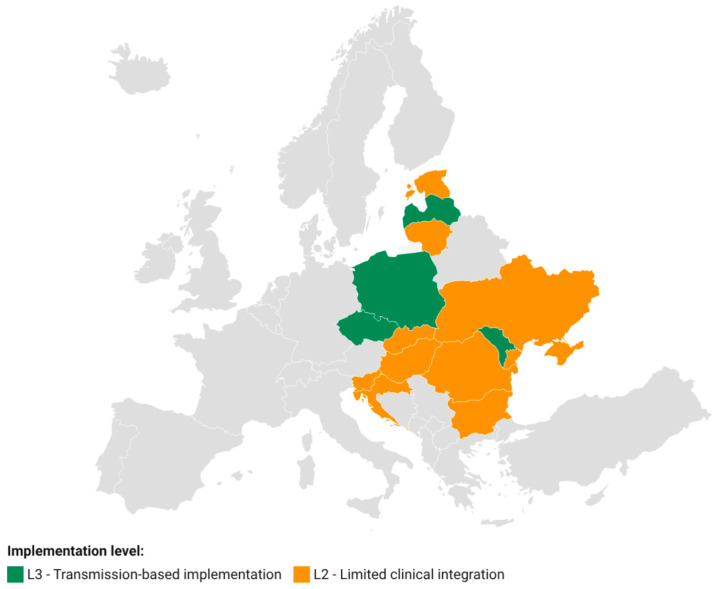
Genomic implementation maturity (L0–L4) across Central and Eastern Europe. Countries are classified according to the highest implementation level achieved based on the predefined maturity framework (L2, limited clinical integration; L3, repeated use for transmission and clustering analyses). Grey indicates countries outside the predefined study region.

The geographic distribution of implementation maturity levels is illustrated in Figure 2.

### 3.4. Models for the Use of Genomic Sequencing

In the 28 studies included, genomic sequencing was predominantly used for drug resistance prediction and transmission analysis, with varying degrees of integration into routine surveillance.

1. Resistance prediction;
  Genomic prediction of drug resistance was reported in 25 of 28 studies, representing the most common application of sequencing in the region. In most cases, analyses were based on the identification of resistance-associated mutations using validated catalogs and dedicated bioinformatics tools. The drugs evaluated mainly included first- and second-line treatments, and in some cases, new or repurposed drugs were also analyzed.
2. Transmission analysis and genomic clustering;
  Transmission analysis using SNP-based methods or phylogenetic reconstruction was reported in 19 of 28 studies. The thresholds used to define genomic clusters varied between studies, and a significant proportion did not explicitly specify an SNP cut-off. The use of transmission analysis was more common in countries classified as L3, where sequencing was repeatedly applied to investigate clusters and reconstruct epidemiological dynamics.
3. Monitoring resistance to new or repurposed drugs;
  Assessment of resistance to drugs such as bedaquiline, linezolid, clofazimine, or delamanid was reported in 8 of 28 studies, predominantly in countries with more advanced levels of genomic implementation. This use reflects the gradual expansion of WGS applicability beyond standard prediction of resistance to classic drugs.
4. Internal capacity versus outsourcing;
  Internal capacity to perform WGS was documented in several countries, while other countries used sequencing through external collaborations or international infrastructures. The presence of sustained internal capacity was associated with repeated use for transmission analysis and higher levels of implementation maturity. In the absence of an operational national infrastructure, the use of sequencing has remained predominantly focused on resistance prediction and dependent on projects or collaborations.

Usage patterns varied according to the level of implementation maturity, highlighting differences between countries classified at level L2 and those classified at level L3. A comparative summary between countries classified at levels L2 and L3 is presented in Table 3.

### 3.5. Monitoring Resistance to New and Repurposed Drugs

In the 28 studies included, the assessment of resistance to new or repurposed drugs was reported in 8 studies (28.6%), representing an emerging but still limited component of genomic applications in the region. The drugs analyzed mainly included bedaquiline (BDQ), linezolid (LZD), delamanid (DLM), and clofazimine (CFZ), with the number of studies reported for each molecule not being mutually exclusive, as some papers evaluated multiple drugs in parallel.

BDQ was the most frequently reported drug, being analyzed in all studies that included the evaluation of resistance to new or reused therapies. Linezolid, delamanid, and clofazimine were reported progressively less frequently. Genomic analyses aimed to identify resistance-associated mutations in genes known for these molecules, mainly including Rv0678 (mmpR5), atpE, and pepQ for BDQ/CFZ, rrl and rplC for LZD, and genes involved in nitroimidazole activation (e.g., ddn, fgd1, fbiA–C) for DLM. However, reporting of genetic markers was heterogeneous across studies, with no uniform regional standardization of the genes analyzed or the criteria used for interpreting variants.

Studies evaluating these drugs came from diverse national and multicenter contexts, including both L2 and L3 countries. In general, genomic monitoring of new drugs was associated with more established genomic infrastructure or participation in international projects.

A summary of the distribution of drugs evaluated is presented in Table 4.

### 3.6. Clustering and SNP Thresholds

Genomic transmission and clustering analysis was reported in 19 of the 28 included studies. Of these, most used approaches based on SNP differences or phylogenetic reconstructions, and only one study applied non-SNP clustering (classical genotyping, VNTR-based).

The thresholds used to define genomic clusters varied considerably between studies. The most common threshold was ≤5 SNPs (*n* = 6), generally associated with recent transmission. Other reported thresholds included ≤12 SNPs (*n* = 3), ≤10 SNPs (*n* = 2), and <13 SNPs (*n* = 1). One study used wider phylogenetic distances (approximately 20–40 SNPs), reflecting a more exploratory approach to genetic relationships.

In two studies, the SNP threshold was not clearly defined, and in three studies, information on the threshold used was not explicitly reported.

This methodological heterogeneity highlights the lack of regional standardization in defining genomic transmission in the context of MDR-TB.

A summary of the thresholds used is presented in Table 5.

### 3.7. Molecular Epidemiology and Lineage Distribution

Molecular epidemiological analysis of the distribution of *Mycobacterium tuberculosis* lineages was reported in several of the included studies, but the level of detail varied considerably between papers.

Lineage 2 (Beijing) was mentioned in 9 studies (32.1%), representing the most frequently reported genetic lineage in the studies analyzed, particularly in the context of MDR-TB. In several cases, it was associated with clustering analyses or the description of dominant clades.

Lineage 4 (Euro-American) and Lineage 3 (East-African Indian) were each reported in 5 studies (17.9%), and Lineage 1 (Indo-Oceanic) in 3 studies (10.7%).

Five studies (17.9%) reported clade-specific analyses, including dominant subclades, temporal phylogenetic reconstructions (TMRCA), or descriptions of the emergence of epidemic clusters.

The reporting of lineage distribution was not uniform across studies, and phylogenetic classification methodologies varied.

A summary of the reported lineage distribution is presented in Table 6.

## 4. Discussion

### 4.1. Principal Findings

This scoping review highlights that CEE countries have reported the use of WGS and have developed varying degrees of operational capacity for the use of WGS in the context of MDR-TB, but the formal integration of this technology into national surveillance systems remains incomplete. This gap means that genomic tools are not yet fully used to support disease control and transmission monitoring.

All 13 countries assessed fell within intermediate maturity levels (L2–L3), without meeting the predefined criteria for an L4 level of integrated genomic surveillance (Table 2). The predominant use of WGS for genomic prediction of drug resistance (25 of 28 studies) (Table 1) suggests that immediate clinical applicability was the main driver of implementation, rather than prospective integration into institutionalized surveillance mechanisms. This suggests that WGS is mainly used for individual patient management, with more limited use at the population level.

Transmission analysis was reported in 19 of 28 studies, but the SNP thresholds used varied considerably, reflecting the absence of regional methodological harmonization. Monitoring of resistance associated with new or repurposed drugs was less prevalent (8 of 28 studies), and reporting of genetic markers was heterogeneous, suggesting an emerging but still non-standardized stage of implementation. These differences make it more difficult to compare results between countries and to use the data in a coordinated way.

Overall, the data suggest that the region is in a transitional phase between the development of technical capacity and the systemic integration of genomic surveillance, a situation comparable to previous observations at the European level in WGS-based pilot surveillance initiatives and in recent assessments of molecular implementation at the EU/EEA level [4,36]. This stage is particularly relevant in regions with a high burden of MDR-TB, where improved surveillance and transmission monitoring are essential.

### 4.2. Regional Maturity Pattern: The Intermediate Implementation Plateau

The exclusive distribution of countries assessed at levels L2 and L3 suggests the existence of a coherent regional pattern of intermediate implementation of WGS for MDR-TB. The absence of L0 and L1 levels indicates that the use of genomic sequencing is no longer limited to exploratory stages or isolated pilot projects, but reflects reported use and emerging technical capacity across the region based on published evidence (Table 2).

At the same time, the absence of an L4 level of integrated genomic surveillance reflects that the systemic integration of WGS into NTP has not been institutionally formalized. This contrast between technological availability and limited structural integration suggests the existence of an “intermediate plateau” of implementation, characterized by operational use without full institutionalization.

Level L2 was defined by recurrent use for resistance prediction, but without prospective integration into national surveillance workflows or standardized reporting mechanisms. Level L3 reflected a more advanced degree of use, including repeated transmission analyses and more consistent reporting of bioinformatics pipelines, but without clear evidence of formalization of genomic surveillance at the national policy level.

Similar observations regarding the gap between technical capacity and institutional integration have previously been reported in the European pilot initiative coordinated by the ECDC, which demonstrated the feasibility of harmonized WGS surveillance but highlighted the need to strengthen integrated national systems for prospective implementation [4]. Previous assessments of the use of molecular and genomic typing in EU/EEA countries have also highlighted significant heterogeneity in the integration of genomic data into routine surveillance and the lack of standardized procedures for cross-border collaboration [37]. Recent reports on the status of molecular surveillance in the post-pilot period confirm the persistence of variations in WGS coverage and the level of institutional integration between Member States [38]. These variations reflect not only differences in technical capacity, but also systemic constraints reported at the European level, including difficulties in bioinformatics standardization, limitations in cross-border data exchange infrastructure, and the lack of sustainable funding mechanisms for routine genomic surveillance.

Therefore, the L2–L3 model identified in this analysis does not indicate an absence of regional capacity, but rather a transitional stage between technological adoption and the formal, institutionalized integration of genomic surveillance into national tuberculosis control programs.

### 4.3. Genomic Resistance Prediction as the Dominant Use Case

Genomic prediction of drug resistance was the most frequently implemented application of WGS in the included studies, being reported in 25 of 28 publications (Table 1). This predominance suggests that immediate clinical utility—rather than prospective integration into institutionalized surveillance mechanisms—is the main driver of WGS implementation in the region.

The ability of WGS to rapidly identify resistance-associated mutations and extend drug susceptibility profiling beyond the limits of conventional phenotypic testing is well-documented in European reference laboratories. Data from EU/EEA countries indicate that the implementation of WGS has significantly improved the performance and scale of resistance testing, particularly for multidrug-resistant tuberculosis [39]. Similarly, the pilot initiative coordinated by the ECDC has demonstrated the feasibility of applying standardized genomic resistance prediction workflows in multiple national contexts, highlighting the interoperability potential of this approach [4].

At the international level, recent recommendations from the World Health Organization support the progressive integration of advanced molecular methods into tuberculosis resistance diagnosis, emphasizing their role in expanding and accelerating resistance detection, including for second-line drugs and new molecules [40]. This position reinforces the status of genomic resistance prediction as the functional core of WGS adoption.

However, the existence of an operational workflow for genomic resistance prediction as reported in the included studies is not equivalent to formal integration into NTP. In several contexts analyzed, WGS appears to operate in parallel with conventional testing, often within research initiatives or externally funded projects, with no clear evidence of systemic integration into routine surveillance. This gap between technological availability and institutional integration supports the characterization of the region as being in an intermediate stage of implementation.

Overall, the data suggest that genomic resistance prediction is the fundamental layer of WGS adoption in Central and Eastern Europe, preceding broader structural integration into the national surveillance architecture.

### 4.4. Transmission Analysis and SNP Threshold Heterogeneity

WGS-based transmission analysis was reported in 19 of the 28 included studies, indicating relatively widespread use of genomic data for cluster investigation and phylogenetic relationship reconstruction. The distribution of thresholds used to define genomic clusters is summarized in Table 5. However, methodological approaches and thresholds used to define genomic clusters varied considerably between studies, reflecting the absence of a clear regional consensus.

The most frequently reported threshold was ≤5 SNPs, generally used as an indicator of recent transmission. Other studies adopted broader thresholds (≤10, ≤12, or <13 SNPs), and in some cases, the threshold was not explicitly specified. This methodological variability limits direct comparability between studies and complicates the aggregate interpretation of transmission dynamics at the regional level.

The international literature emphasizes that defining a universally applicable SNP threshold is complex, as it depends on the epidemiological context, the population structure of circulating strains, and the technical parameters of bioinformatic analysis [41]. Although WGS offers superior resolution compared to classical genotyping methods, the interpretation of genetic distances requires methodological standardisation to ensure cross-border comparability and consistency of epidemiological surveillance [42].

European harmonization initiatives have demonstrated the feasibility of using standardized analytical workflows, but the uniform adoption of thresholds and criteria for defining clusters remains incomplete [4]. In the absence of such standardization, transmission analysis risks remaining dependent on local frameworks or individual research protocols.

As a result, although WGS is used to investigate transmission in most of the countries analyzed, the heterogeneity of SNP thresholds and reporting practices indicates a level of methodological maturity that is still consolidating, consistent with the intermediate regional profile (L2–L3) identified in this analysis based on the available published evidence.

### 4.5. Monitoring of New and Repurposed Drugs

The assessment of resistance associated with new or repurposed drugs was reported in 8 of the 28 included studies, reflecting an emerging but still limited use of WGS in this field (Table 4). The drugs analyzed mainly included bedaquiline, linezolid, delamanid, and clofazimine, with considerable variation in reporting frequency and analytical approach.

Although the expansion of WGS use to monitor resistance to these molecules suggests a progressive maturation of genomic capacity, reporting of genetic markers and variant interpretation strategies was heterogeneous across studies (Table 4). In several cases, analyses were exploratory or integrated into research contexts, with no indication of systematic use in routine surveillance.

Internationally, the importance of monitoring resistance to new and repurposed drugs is highlighted in recent recommendations on the management of resistant tuberculosis, which emphasize the need for early detection of resistance-associated mutations to prevent the compromise of modernized therapeutic regimens [40]. At the same time, data from European WGS implementation initiatives indicate that the inclusion of these drugs in genomic analysis panels remains variable across countries, reflecting differences in infrastructure, bioinformatics expertise, and clinical integration [39]. The complexity of tuberculosis pathophysiology, including systemic complications such as hypercoagulability, further supports the need for integrated and comprehensive approaches to disease monitoring and management [43].

In this context, the relatively low proportion of studies reporting genomic monitoring of resistance to new drugs in the region analyzed suggests that this field is in a consolidation phase. The systematic integration of the analysis of variants associated with these molecules could be an additional indicator of the transition from the predominantly clinically reactive use of WGS to more complex and prospective genomic surveillance as reflected in the available published studies.

### 4.6. Implications for Structured National Integration

The identified regional L2–L3 model (Table 2) highlights that the current challenge is no longer technological adoption, but rather the coherent institutional integration of genomic flows into national surveillance systems. The data analyzed indicate that most countries have reported the use of WGS and varying degrees of operational capacity, but institutional integration remains incomplete. This gap limits the practical use of genomic data for MDR-TB surveillance and control, particularly in complex clinical contexts such as TB coinfections, which further complicate diagnosis and management [44].

A first structural direction derived from these findings is the need for minimum analytical standardization, including explicit reporting of SNP thresholds and bioinformatic pipelines used. The lack of methodological harmonization limits international comparability and may affect the epidemiological interpretation of clusters, an issue also highlighted in European genomic surveillance initiatives [4]. Without this level of standardization, genomic data remain difficult to use in a consistent way across countries.

Secondly, strengthening the link between genomic resistance prediction and clinical decision-making is an essential element of the transition to a higher level of maturity. Although resistance prediction is widely used, its formal integration into national therapeutic algorithms and programmatic reporting mechanisms remains variable, a situation also observed in recent European assessments of WGS implementation [38]. This limits the impact of genomic data on patient management and treatment decisions.

A third element is the development of a prospective framework for monitoring resistance to new and repurposed drugs, an area still in its infancy in the region (Table 4). In the context of the expanding use of modern regimens for MDR-TB, the systematic integration of genomic analyses associated with these molecules may be an indicator of the maturation of surveillance. Improved monitoring of resistance to these drugs is important for preserving their effectiveness.

Finally, the relationship between the existence of internal sequencing capacity and the level of maturity identified suggests that local infrastructure and operational continuity are determining factors in progress toward systemic integration. Countries with repeated use and consolidated internal capacity tend to achieve more advanced levels of epidemiological application (Table 2), without, however, documenting full formal integration at the national level. Strengthening local capacity may therefore support more stable and effective surveillance systems.

These findings indicate that the transition from predominantly technical use of WGS to institutionalized genomic surveillance requires not only technological availability, but also methodological standardization, clinical integration, and explicit programmatic mechanisms. Together, these elements are necessary to support more effective control of MDR-TB at the population level.

### 4.7. Strengths and Limitations

This analysis provides a structured regional synthesis of genomic sequencing implementation for MDR-TB in Central and Eastern Europe. It uses a predefined maturity framework (L0–L4) that was applied consistently across all included countries. A key strength of the study is the explicit definition of the classification criteria—internal technical capacity, use for resistance prediction, transmission analysis, and programmatic integration. These criteria allowed for a coherent comparison between countries with different epidemiological and institutional contexts.

In addition, sample size was defined consistently as the number of isolates analyzed molecularly. Genomic cohorts were also verified directly from the original publications. This helped reduce discrepancies between clinical cohorts and the subsets analyzed by WGS. It also improved internal numerical consistency and limited the risk of overestimating the level of implementation.

Another strength is the clear geographic focus on a region with a specific epidemiological profile. This made it possible to identify a shared regional pattern of implementation (L2–L3), which might not have been evident in a broader and more heterogeneous global analysis.

However, several limitations should be acknowledged. First, the search was limited to published literature indexed in PubMed and Web of Science. As a result, some genomic activities that remain unpublished or internal implementations that have not been formally reported may have been missed. Second, the classification of maturity levels relied only on information reported in the included articles. Institutional documents, national guidelines, or unpublished policy data were not included in the analysis, which may result in a partial underestimation of implementation capacity. Third, data extraction and study selection were performed by a single reviewer, which may introduce a degree of subjectivity, although a predefined framework and standardized criteria were applied throughout the process.

Finally, the methodological heterogeneity of the included studies—particularly regarding SNP thresholds and bioinformatic strategies—did not allow for a quantitative meta-analysis. For this reason, the study intentionally follows a descriptive and mapping approach consistent with scoping review methodology. In addition, the findings of this review reflect the available published literature up to the time of the search and should be interpreted as an overview of reported implementation rather than a real-time assessment of current national capacity.

### 4.8. Overall Interpretation

Overall, the results of this analysis indicate that Central and Eastern Europe is currently in an intermediate stage of genomic sequencing implementation for MDR-TB. Evidence from the included studies suggests that technical capacity is generally available, but institutional integration remains incomplete. Genomic resistance prediction is widely used, and WGS-based transmission analysis is reported in most countries. However, formal integration of genomic surveillance within NTP is still limited. This means that genomic data are not yet fully used to support routine surveillance and disease control.

The regional L2–L3 pattern identified in this study suggests a transitional phase between technological adoption and systemic integration. In this phase, infrastructure, methodological standardization, and mechanisms for clinical integration are likely to determine further progress toward more advanced levels of implementation. Progress in these areas will be important for improving surveillance and understanding transmission of MDR-TB. The absence of an L4 level in the region should be interpreted as reflecting the current state of reported implementation rather than confirmed absence or presence of full national capacity.

The region therefore appears to be at an important stage in the development of genomic surveillance for MDR-TB. Strengthening programmatic structures and improving analytical harmonization could help transform existing technical capacity into a fully integrated public health surveillance tool.

### 4.9. Future Directions

Future progress in the implementation of genomic sequencing for MDR-TB in Central and Eastern Europe will depend on several key developments. First, the establishment of minimum methodological standards, including harmonized SNP thresholds and standardized bioinformatic pipelines, is essential to improve comparability across countries.

Second, stronger integration of WGS into national tuberculosis programs is needed to ensure that genomic data are systematically incorporated into surveillance and clinical decision-making processes.

Third, the expansion of genomic monitoring for resistance to new and repurposed drugs represents an important step toward more comprehensive surveillance, particularly in the context of evolving treatment regimens.

Finally, the development of sustainable local sequencing capacity and infrastructure will be critical to support continuous and programmatic use of WGS beyond research-based or externally funded initiatives.

## 5. Conclusions

The implementation of genomic sequencing for MDR-TB in Central and Eastern Europe reflects an intermediate stage of development, in which evidence from published studies suggests that technical capacity is increasingly available, but institutional integration remains partial. From a public health perspective, this indicates that the potential of genomic tools to improve MDR-TB control and surveillance has not yet been fully realized. The use of WGS is still predominantly clinical and precedes the establishment of a formalized and harmonized system of genomic surveillance at the regional level.

Identifying this shared pattern of implementation provides a useful analytical framework for evaluating future progress and for comparing national developments over time. These findings are relevant for strengthening infectious disease surveillance strategies and for guiding the integration of genomic approaches into national tuberculosis control programs. The transition toward fully integrated genomic surveillance will depend on transforming existing technical capacity into a stable and standardized programmatic mechanism.

## Figures and Tables

**Table 1:** Characteristics of included studies (*n* = 28).

No.	First Author (Year)	Country	Study Context	Sample Size ^1^	Study Period	Sequencing Type	Platform	Pipeline Reported	Transmission Analysis	Genomic Resistance Prediction	New Drugs Evaluated
1	Jagielski (2015) [10]	Poland	Retrospective surveillance	46	2004	Classical molecular typing + targeted Sanger sequencing	ABI PRISM 3130xl (Sanger)	No	Yes	Yes	No
2	Brown (2015) [9]	Lithuania, United Kingdom	Methodological/pilot implementation	24	Not specified	WGS (direct sputum-based)	Illumina MiSeq	Partial	No	Yes	No
3	Fiebig L (2017) [11]	Romania, Austria, Germany	Outbreak investigation	10	2010–2014	WGS (culture-based)	Illumina MiSeq	Partial	Yes	Yes	No
4	Popovici O (2018) [12]	Romania	Outbreak investigation	7	2015–2018	WGS (culture-based)	Illumina MiSeq	Yes	Yes	Yes	No
5	Ruesen C (2018) [3]	Romania	Pilot implementation	72	2014–2016	WGS (culture-based)	Illumina NextSeq	Yes	No	Yes	No
6	Sinkov V (2018) [14]	Republic of Moldova, Belarus, Russia	Molecular epidemiology	149	Not specified	WGS (culture-based)	Illumina platform	Yes	Yes	Yes	No
7	Daum LT (2018) [13]	Ukraine	Isolate characterization	75	2016	Targeted NGS (drug-resistance gene panel)	Illumina MiSeq	Yes	No	Yes	No
8	Daum LT (2019) [15]	Ukraine	Isolate characterization	91	2016	WGS (culture-based)	Illumina MiSeq	Yes	No	Yes	No
9	Wollenberg K (2020) [16]	Republic of Moldova	Retrospective surveillance	278	2007	WGS (culture-based)	Illumina platform	Yes	Yes	Yes	No
10	Pole I (2020) [19]	Latvia	Molecular epidemiology	908	2008–2012	Classical molecular typing (MIRU-VNTR/spoligotyping)	PCR-based typing	No	Yes	No	No
11	Merker M (2020) [18]	Ukraine	Molecular epidemiology	177	2015	WGS (culture-based)	Illumina MiSeq/NextSeq	Yes	Yes	Yes	No
12	Bitar (2020) [17]	Czechia	Isolate characterization	40	2005–2017	WGS (culture-based)	Illumina MiSeq	Yes	No	Yes	No
13	Dohál M (2021) [26]	Slovakia	Molecular epidemiology	12	2018–2019	WGS (culture-based)	Illumina MiSeq	Yes	Yes	Yes	Yes
14	Brown TS (2021) [22]	Republic of Moldova	Molecular epidemiology	283	2013–2014	WGS (culture-based)	Illumina MiSeq	Yes	Yes	Yes	No
15	Sanchini A (2021) [21]	Multiple countries (public WGS repository)	Molecular epidemiology	1335	1996–2013	WGS (culture-based)	Illumina platform	Yes	Yes	Yes	No
16	Mokrousov I (2021) [20]	Estonia	Molecular epidemiology	278	1999–2015	Classical molecular typing (non-WGS genotyping)	PCR-based typing	No	No	Yes	No
17	Yang C (2022) [27]	Republic of Moldova	Prospective genomic surveillance	1834	2018–2019	WGS (culture-based)	Illumina platform	Yes	Yes	Yes	Yes
18	Yordanova S (2022) [24]	Bulgaria	Pilot implementation	65	2017–2019	WGS (culture-based)	Illumina platform	Yes	Yes	Yes	Yes
19	Dohál M (2022) [23]	Czechia	Molecular epidemiology	65	2005–2020	WGS (culture-based)	Illumina platform	Yes	Yes	Yes	Yes
20	Chesov (2022) [25]	Republic of Moldova	Cross-border analysis	97	2016–2018	WGS (culture-based)	Illumina NextSeq	Yes	No	Yes	Yes
21	Viksna A (2023) [30]	Latvia	Molecular epidemiology	63	2012–2014	WGS (culture-based)	Illumina platform	Yes	No	Yes	Yes
22	Noroc E (2023) [29]	Republic of Moldova	Molecular epidemiology	268	2014–2015	WGS (culture-based)	Illumina platform	Yes	Yes	No	No
23	Bakuła Z (2023) [28]	Poland	Molecular epidemiology	89	2018–2021	Classical molecular typing (MIRU-VNTR/spoligotyping)	PCR-based typing	Yes	Yes	No	No
24	Viksna A (2024) [31]	Latvia	Molecular epidemiology	80	2002–2019	WGS (culture-based)	Illumina MiSeq	Yes	No	Yes	No
25	Dohál M (2024) [32]	Czechia, Slovakia	Prospective genomic surveillance	91	2021–2022	WGS (culture-based)	Illumina platform	Yes	Yes	Not reported	No
26	Minias A (2025) [34]	Poland	Molecular epidemiology	163	2003–2020	WGS (culture-based)	Illumina NextSeq	Yes	Yes	Yes	No
27	Saluzzo F (2025) [33]	Italy, Romania, United Kingdom	Cross-border analysis	44	2016–2025	WGS (culture-based; outbreak cluster snpCL1)	Illumina platform	Yes	Yes	Yes	Yes
28	Vasiliauskaitė L (2026) [35]	Lithuania	Molecular epidemiology	66	2016–2023	WGS (culture-based)	Illumina NovaSeq	Yes	Yes	Yes	Yes

^1^ Sample size refers to the number of *Mycobacterium tuberculosis* isolates included in the final molecular analysis after quality filtering. It does not represent the total number of enrolled patients or screened samples. Abbreviations: WGS, whole-genome sequencing; Targeted NGS, next-generation sequencing of selected resistance-associated genes; Classical molecular typing includes MIRU-VNTR, spoligotyping, IS6110-RFLP or other PCR-based genotyping methods. Illumina platform refers to MiSeq, NextSeq, NovaSeq or other Illumina instruments when the exact model was not specified in the original publication.

**Table 2:** Genomic implementation maturity (L0–L4) across Central and Eastern European countries included in the review.

Country	No. of Studies	Internal WGS Capacity	Transmission Analysis	New Drugs Evaluated	Highest Level Achieved
Poland	3	Yes (recent)	Yes	No	L3
Czechia	2	Yes	Yes	Yes	L3
Latvia	3	Yes	Yes	No	L3
Republic of Moldova	4	No (outsourced)	Yes	Yes	L3
Lithuania	2	Yes	Limited	Yes	L2
Ukraine	3	Partial	Limited	Yes	L2
Slovakia	2	Partial	Limited	No	L2
Hungary	1	No	Limited	No	L2
Romania	4	No (outsourced)	Limited (outsourced)	Yes	L2
Bulgaria	2	Partial	Limited	No	L2
Croatia	1	No	No	No	L2
Slovenia	1	No	No	No	L2
Estonia	1	Partial	Limited	No	L2

Abbreviations: WGS, whole-genome sequencing; Implementation levels (L0–L4): L0, no documented genomic capacity; L1, research-only implementation; L2, repeated use for resistance prediction with limited clinical integration; L3, use for transmission and clustering analyses; L4, formally integrated national genomic surveillance within the NTP.

**Table 3:** Genomic sequencing utilization patterns stratified by implementation maturity level.

Application Domain	L2 Countries (*n* = 9)	L3 Countries (*n* = 4)
Primary use of sequencing	Resistance prediction	Resistance prediction + transmission analysis
Transmission/clustering analysis	Absent, limited or externally supported	Systematic and recurrent
SNP threshold reporting	Inconsistently reported	More frequently defined
New or repurposed drug resistance monitoring	Rare	Frequently reported
Internal WGS capacity	Often externalized or project-based	Frequently internally established
Integration within NTP	Not formally integrated	Selectively integrated, without formal national policy

Classification based on the country-level maturity model (L0–L4) defined in the Section 2 (Material and Methods).

**Table 4:** Monitoring of resistance to new and repurposed anti-tuberculosis drugs across included studies (*n* = 28).

Drug	Number of Studies Reporting	Percentage (%)	Genomic Resistance Markers Evaluated *	Phenotypic Confirmation Reported
Bedaquiline (BDQ)	8	28.6	Efflux-related (e.g., Rv0678), atpE-associated mutations	Reported variably
Linezolid (LZD)	7	25	Ribosomal gene mutations (e.g., rrl, rplC)	Reported variably
Delamanid (DLM)	6	21.4	Nitroimidazole activation pathway genes	Limited reporting
Clofazimine (CFZ)	5	17.9	Efflux-related mutations (e.g., Rv0678)	Rarely specified

* Percentages calculated from total included studies (*n* = 28). Specific genes varied between studies; markers are summarized at pathway/gene-class level.

**Table 5:** SNP thresholds and clustering approaches reported in transmission analyses (*n* = 19 studies with transmission analysis).

Clustering Approach/SNP Threshold	Number of Studies
≤5 SNP	6
≤12 SNP	3
≤10 SNP	2
<13 SNP	1
Phylogenetic distance (~20–40 SNP)	1
Not clearly defined	2
Not reported	3
Non-SNP clustering (VNTR-based)	1

**Table 6:** Reported *Mycobacterium tuberculosis* lineages and molecular epidemiology analyses across included studies (*n* = 28).

Molecular Epidemiology Feature	Number of Studies Reporting	Percentage of Total Studies (%)
Lineage 2 (Beijing)	9	32.1
Lineage 4 (Euro-American)	5	17.9
Lineage 3 (East-African Indian)	5	17.9
Lineage 1 (Indo-Oceanic)	3	10.7
Clade-specific phylogenetic analysis (subclades/TMRCA/epidemic clades)	5	17.9

Percentages calculated from total included studies (*n* = 28). Abbreviations: TMRCA, time to the most recent common ancestor.

## Data Availability

No new datasets were generated or analyzed during this study. All data used in this review are available in the published literature cited in the reference list.

## Supplementary Materials

PRISMA-ScR Checklist used for reporting of this scoping review. The following supporting information can be downloaded at https://www.mdpi.com/article/10.3390/diseases14050172/s1.

## Abbreviations

The following abbreviations are used in this manuscript:

BDQ	bedaquiline
CFZ	clofazimine
DLM	delamanid
DST	drug susceptibility testing
LZD	linezolid
MDR-TB	multidrug-resistant tuberculosis
MIC	minimum inhibitory concentration
NGS	next-generation sequencing
NTP	National Tuberculosis Program
PRISMA-ScR	Preferred Reporting Items for Systematic Reviews and Meta-Analyses extension for Scoping Reviews
RR-TB	rifampicin-resistant tuberculosis
SNP	single nucleotide polymorphism
TB	tuberculosis
TMRCA	time to the most recent common ancestor
WGS	whole-genome sequencing
XDR-TB	extensively drug-resistant tuberculosis
